# Pooled analysis of the comparative efficacy between tacrolimus and infliximab for ulcerative colitis

**DOI:** 10.1097/MD.0000000000011440

**Published:** 2018-08-10

**Authors:** Yi-Juan Liu, Hua Fan, Wei-Wei Zhen, Xing Yu, Jin-Tong Chen, Cheng-Dang Wang

**Affiliations:** Department of Gastroenterology, The First Affiliated Hospital, Fujian Medical University, Fuzhou, China.

**Keywords:** infliximab, tacrolimus, ulcerative colitis

## Abstract

**Background:**

Acute moderate-to-severe steroid-refractory ulcerative colitis (UC) has a poor prognosis and requires optimal rescue therapy. A pooled analysis was conducted to assess tacrolimus and infliximab (IFX) as rescue agents in patients with moderate-to-severe and steroid-refractory UC.

**Methods:**

A literature search identified studies that investigated tacrolimus and IFX in moderate-to-severe steroid-refractory patients with UC. The primary outcome was short-term clinical response to treatment, including the remission and response rates. Secondary outcomes included the rates of colectomy at 3 months and adverse events rate.

**Results:**

A total of 6 studies comprising 438 cases were eligible for inclusion. The pooled analysis showed that the short-term clinical response rate, clinical remission rate, and 3-month colectomy rate were 72.1%, 52.4%, and 10.1%, respectively, for those receiving tacrolimus, and 76.9%, 48.8%, and 12.4%, respectively, for those receiving IFX. No significant difference was, however, seen for tacrolimus compared with IFX with regard to clinical remission rate (odds ratio [OR] =1.08, 95% confidence interval [CI] = 0.77–1.49, *P* = .67), clinical response rate (OR = 0.92, 95% CI = 0.63–1.34, *P* = .66), and 3-month colectomy rate (OR = 0.86, 95% CI = 0.39–1.93, *P* = .72). More adverse events were, however, observed in the Tac group (OR = 2.16, 95% CI = 1.25–3.76, *P* = .006).

**Conclusions:**

Our meta-analysis suggested that both tacrolimus and IFX appeared to be effective and safe for the rescue therapy of moderate-to-severe active UC and steroid-refractory UC. Therefore, tacrolimus is another choice for these patients.

## Abstract 2

Supplemental Digital Content is available in the text

## 1 Introduction

Ulcerative colitis (UC) comprises a range of chronic relapsing inflammatory bowel diseases of unknown etiology. Among them, up to 25% are acute severe UC.^[1]^ Corticosteroids remain the first-line treatment; however, up to 30% of the patients are unresponsive to corticosteroid therapy and will require salvage therapy.^[2]^ Both calcineurin inhibitors and antitumor necrosis factor alpha (TNFα) antibodies have been considered for salvage therapy.^[3,4]^ Medical salvage therapies for these patients can help to avoid colectomy and can improve long-term outcomes.

Ciclosporin (CsA) was the first shown to be effective in acute severe steroid-refractory UC, with short-term response rates ranging from 64% to 82%.^[5,6]^ It is, however, associated with significant adverse events, including opportunistic infections and nephrotoxicity; therefore, its use in UC is limited to induction therapy for acute moderate-severe disease. Tacrolimus (Tac) is a newly developed calcineurin inhibitor. The utility of Tac to treat steroid-refractory UC has been reported, with short-term response rates ranging from 55% to 98%, with less severe adverse events than CsA.^[7–9]^ Therefore, Tac is now regarded as one of the main therapeutic options for steroid-refractory UC. Infliximab (IFX) is a TNFα antagonist, whose efficacy has been established in randomized controlled trials (RCTs). IFX was superior to placebo in achieving clinical remission and response, mucosal healing, and colectomy-free survival in patients with moderate-to-severe active UC.^[10,11]^ It is, however, also associated with potentially severe adverse events, such as infusion reactions and infections.

The mechanisms of action of calcineurin inhibitors and anti-TNF agents are completely different. Many studies have been conducted to determine which medication is more appropriate in patients with refractory UC. Two recent meta-analyses did not reveal any significant differences between IFX and CsA in terms of colectomy and adverse events rates.^[9,12]^ In Komaki et al's study,^[9]^ a network meta-analysis was conducted to evaluate the efficacy of IFX, cyclosporine, and Tac for severe patients with UC, which suggested that IFX was somewhat superior to Tac and cyclosporine. This result was, however, conducted from indirect comparisons, and the number of studies in each meta-analysis was small ranging from 1 to 2, and it was not possible to conclude that IFX, Tac, and cyclosporine have comparable efficacy; therefore, the results from this meta-analysis should be interpreted with caution. There are also some studies that compared the efficacy and safety between Tac and IFX directly in refractory UC^[13–18]^; however, the results were not consistent. Therefore, we undertook this meta-analysis to identify observational studies and clinical trials that compared Tac and IFX as rescue agents in steroid-refractory or acute moderate to severe UC.

## 2 Materials and methods

### 2.1 Ethical statement

Ethical approval was not necessary, because this is a meta-analysis.

### 2.2 Search strategy

A systematic literature search was conducted to identify studies that compared treatment with Tac or IFX in acute severe UC and steroid-refractory UC. Potential studies were identified from Pubmed (1993–November 2017) and Embase (1993–November 2017) with the following keywords: “anti-TNF OR infliximab,” “ulcerative colitis OR UC OR colitis,” and “tacrolimus OR FK506 OR tac.” There were no language restrictions. The reference lists of the included studies and previous systematic reviews were searched manually searched to avoid missing relevant publications.

### 2.3 Inclusion and exclusion criteria

All eligible studies that compared the efficacy between Tac and IFX were selected in this meta-analysis. Studies meeting the following criteria were included: RCTs, open-label prospective, observational studies, cohort, and case-control studies; studies comparing Tac or IFX as rescue therapies with outcomes reported for both cohorts; and subjects were patients with acute moderate to severe UC or steroid-refractory UC. Case reports, letters, reviews, systematic reviews, and meta-analysis were excluded. Reports that provided no sufficient data were also excluded from the meta-analysis.

### 2.4 Data extraction and quality assessment

The following data were extracted from each included study: the first author, publication year, country, design type, number of cases, clinical remission rate, clinical response rate, colectomy rate, adverse events rate. Data extraction was carried out independently by 2 authors, and discrepancies were resolved by consensus in consultation with the third author. The main outcome was the short-term clinical remission rate, clinical response rate, colectomy rate, and the adverse events rate at about 12 weeks. We did not assess the methodological quality of the included studies, given that the quality assessment of observational studies in meta-analysis is controversial (there is only 1 RCT, the other 5 studies were observational studies).

### 2.5 Statistical analysis

Comparisons of Tac or IFX as rescue therapies in UC were assessed by pooled estimates of odds ratio and the 95% confidence interval (CI). *P* values <.05 were considered statistically significant. Heterogeneity was tested using the χ^2^ test and the *I*^2^ test. Fixed-effects models (Mantel-Haenszel) were used when there was no between-study heterogeneity; otherwise, random effect models (DerSimonian and Laird) were used. To assess publication bias, we performed funnel plots and calculated Egger regression; a 2-tailed *P* value <.05 was considered statistically significant. Two authors performed the statistical analysis independently and obtained the same results. Statistical analyses were performed with STATA 11.0 (Stata Corporation, College Station, TX).

## 3 Results

### 3.1 Search results and study characteristics

**Figure 1:**
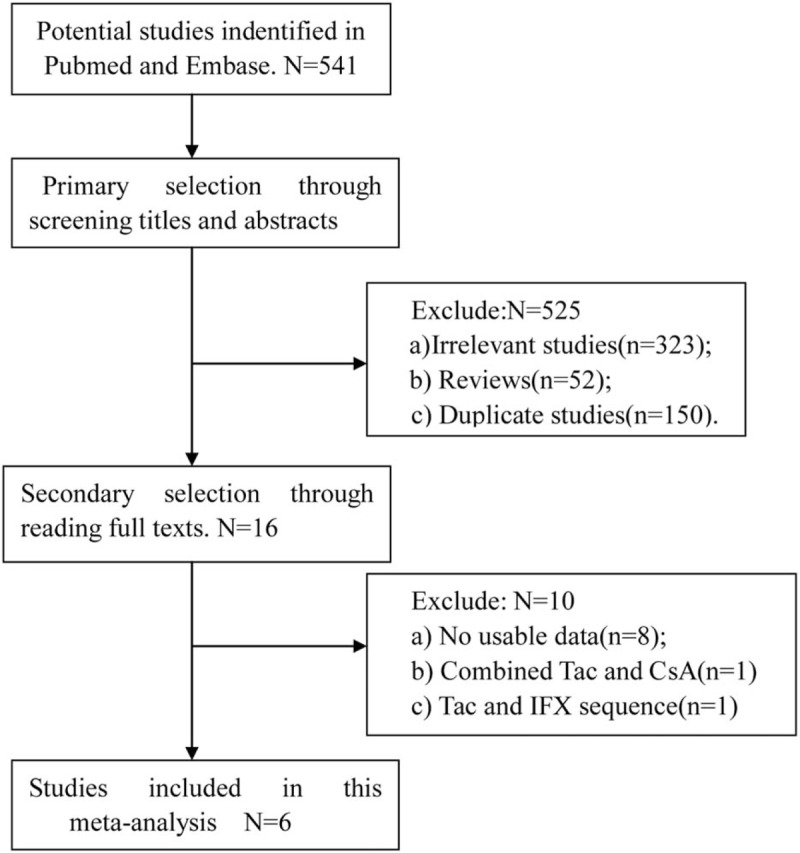
Study selection procedure. CsA = ciclosporin, IFX = infliximab.

Five hundred forty-one potential articles were identified initially using the above-mentioned search strategy, of which 525 were excluded after reading the titles and abstracts. After the reading the full texts, we excluded another 10 studies that had no usable data and did not compare Tac and IFX directly. Finally, 6 studies, which included 438 cases, met the inclusion criteria and were included in this meta-analysis. The detailed study selection procedure is described in Figure 1.

The detailed characteristics of the studies are shown in Table 1. One study was an RCT^[18]^ and the remaining 5 studies were retrospective cohort studies.^[13–17]^ The patients of 2 studies were steroid-refractory UC,^[14,15]^ of 1 study was severe UC,^[13]^ and the other 3 were moderate-to-severe UC.^[16–18]^ The medical treatment in the 6 trials was almost the same. IFX was administered at 5 mg/kg at 0, 2, and 6 weeks at the induction stage, and then as maintenance treatment at 5 mg/kg every 8 weeks. Tac was administered orally at an initial dose of 0.05 to 0.1 mg/kg/day twice a day (the initial dose was 0.025 μg/kg in Nuki's study; however, the whole blood trough level was the same to the other 5 studies), and then the dosage was adjusted to achieve a whole blood trough level of 10 to 15 ng/mL in the initial 2 weeks, and 5 to 10 ng/mL subsequently. The disease activity of UC in the included studies was assessed using different method (2 studies using Mayo score, 2 studies using Clinical Activity Index (CAI) another 2 studies using Ulcerative Colitis Activity Index (UCAI)); therefore, the clinical remission and clinical response were defined using different criteria.

**Table 1:**
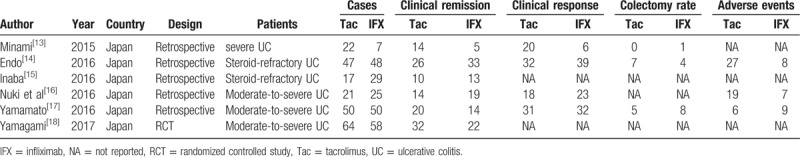
Characteristics of the included studies.

This study was according the PRISMA guidelines (Table 2 and Table 3).

### 3.2 Therapeutic response

**Figure 2:**
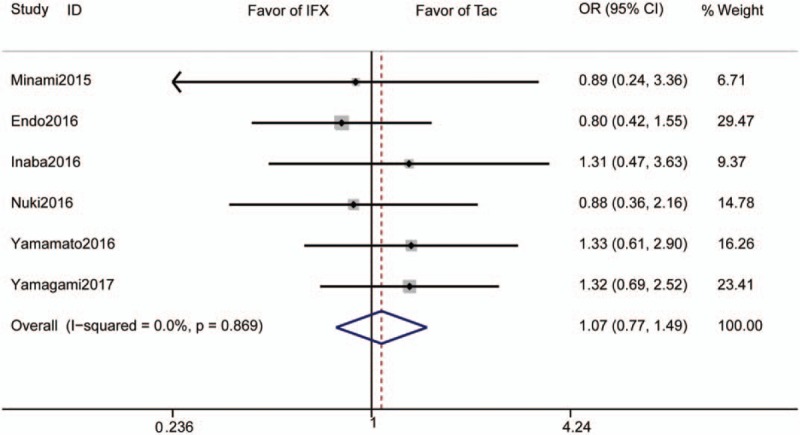
Forrest plot of all studies reporting short-term clinical remission rate. CI = confidence interval, IFX = infliximab, OR = odds ratio.

Six studies reported clinical remission rates and included 221 subjects who received Tac and 217 subjects who received IFX. The pooled clinical remission rate was 52.4% for those receiving Tac and 48.8% for those receiving IFX. The pooled odds ratio (OR) for clinical remission rate was 1.08 (95% CI 0.77–1.49, *P* = .67, Fig. 2).

**Figure 3:**
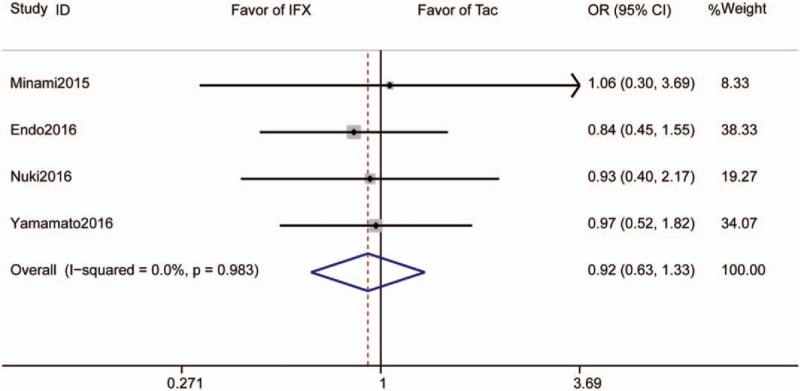
Forrest plot of all studies reporting short-term clinical response rate. CI = confidence interval, IFX = infliximab, OR = odds ratio.

Four studies reported clinical response rates and included 140 subjects who received Tac and 130 subjects who received IFX. The pooled clinical response rate was 72.1% for those receiving Tac and 76.9% for those receiving IFX. The pooled OR for clinical response rate was 0.92 (95% CI 0.63–1.34, *P* = .66, Fig. 3).

### 3.3 Three-month colectomy

**Figure 4:**
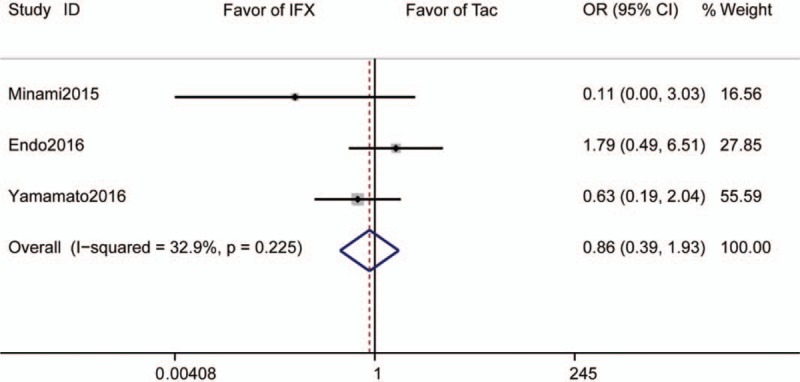
Forrest plot of all studies reporting 3-month colectomy rate. CI = confidence interval, IFX = infliximab, OR = odds ratio.

Three studies reported 3-month colectomy rates and included 119 subjects who received Tac and 105 subjects who received IFX. The pooled colectomy rate was 10.1% for those receiving Tac and 12.4% for those receiving IFX. The pooled OR was 0.86 (95% CI 0.39–1.93, *P* = .72, Fig. 4).

### 3.4 Adverse events rate

**Figure 5:**
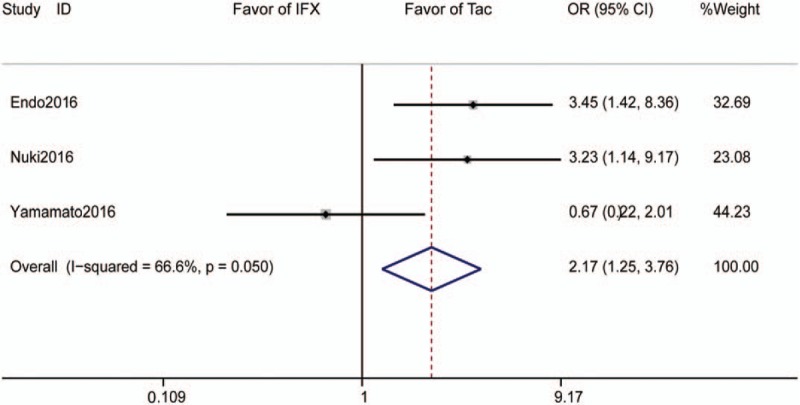
Forrest plot of all studies reporting adverse events rate. CI = confidence interval, IFX = infliximab, OR = odds ratio.

Three studies reported adverse events rate and included 118 subjects who received tacrolimus and 123 subjects who received IFX. The pooled adverse events rate is 44% for those receiving tacrolimus and 19.5% for those receiving IFX. The pooled OR was 2.16 (95% CI 1.25–3.76, *P* = .006, Fig. 5).

### 3.5 Sensitivity analysis and publication bias

**Figure 6:**
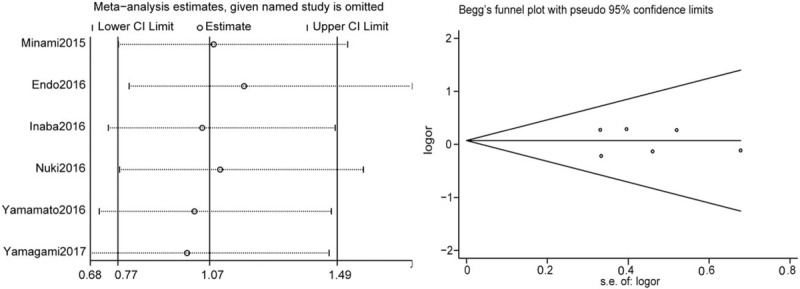
Sensitivity analysis (A) and publication bias (B). CI = confidence interval.

Sensitivity analysis was performed to assess the influence of each individual study on the pooled ORs with regard to the clinical remission rate by sequential omission of individual study. The analysis suggested that no individual study has statistically significant affect on the pooled ORs for the clinical remission rate (Fig. 6A). Potential publication bias was assessed by the Begg test and Egger test. The shapes of the funnel plots were symmetrical and the *P* values were all greater than .05, which indicated no obvious publication bias among these studies regarding the OR for the clinical remission rate (Fig. 6B).

## 4 Discussion

Acute severe steroid-refractory UC is associated with high morbidity and is clinically challenging for physicians and surgeons. An effective and safe rescue therapy is important for those patients to avoid emergent colectomy. Tac/CsA and anti-TNF agents are the most commonly used rescue therapy agents. Although the efficacy of Tac and IFX is well established, few studies have directly compared the efficacy between them. To the best of our knowledge, this is the first meta-analysis to compare the safety and efficacy of Tac and IFX for active UC. Our meta-analysis included 1 RCT and 5 retrospective studies, and found that the short-term clinical response and remission rates were not significantly different between patients treated with TAC and IFX, whereas the adverse events rate was somewhat higher in patients treated with Tac than in those treated with IFX.

The pooled therapeutic response rate of Tac and IFX in this meta-analysis was consistent with previous studies. It is reported that the induction response rates are 61% to 69% for IFX and 62% to 72% for tacrolimus,^[8,19]^ whereas in our meta-analysis, the pooled clinical response rate was 72.1% for those receiving Tac and 76.9% for those receiving IFX; the pooled clinical remission rate is 52.4% for those receiving tacrolimus and 48.8% for those receiving IFX. Although the difference is not statistically significant between Tac and IFX; however, Yamamoto et al's^[17]^ study found that the response rate appeared to be higher in patients treated with tacrolimus in a subgroup analysis restricted to severely active UC, and Minami et al's^[13]^ study found that long-term treatment with Tac rather than IFX could induce a clinically better outcome in patients with severe UC. This finding indicated that Tac tends to be superior in more severely active UC and sheds some light on the use of Tac in these patients; however, large RCTs are needed to confirm this finding.

Our meta-analysis demonstrated relatively high pooled response and remission rates in both the Tac and IFX groups, meaning that a large portion of patients could avoid urgent colectomy and could be treated to achieve remission. Indeed, the pooled short colectomy rate was only 10.1% for those receiving Tac and 12.4% for those receiving IFX. Approximately 50% of patients treated with tacrolimus and 27% to 50% of patients with IFX, however, eventually required colectomy in long-term follow-up.^[20,21]^ Encouragingly, the outcomes of elective colectomy are significantly better than those of urgent colectomy, and should also be considered as a successful outcome of rescue therapy.

In our meta-analysis, the adverse events rate was significantly higher in patients treated with Tac than in those treated with IFX; however, this pooled analysis only included 3 trials, and in Nuki's study, almost all of the patients receiving Tac suffered from hypomagnesemia; therefore, this study influence the pooled results significantly. While excluding Nuki's study, no significant difference was observed between Tac and IFX (OR = 1.57, 95% CI = 0.31–7.84, *P* = .58). Moreover, low magnesium concentrations would not be a reason not to use tacrolimus. In fact, this phenomenon was not reported in other studies. A systematic review examining Tac use in patients with UC reported that the most frequently observed adverse events were tremor and headache, followed by gastrointestinal disorders, and most of the adverse events were mild.^[22]^ In our meta-analysis, the rate of serious adverse events was similar for Tac and IFX, remaining very low, suggesting the safety of Tac and IFX for rescue therapy in UC.

A previous network meta-analysis by Komaki et al^[9]^ was conducted to indirect compare the efficacy and safety of IFX, Tac, and cyclosporine for patients with severe steroid refractory UC. This network meta-analysis demonstrated the rank order of efficacy as IFX, cyclosporine, Tac, and placebo. This study, however, have several limitations: first, the included studies was small, only 2 studies was related with Tac; therefore, the number of studies in each network meta-analysis was extremely small ranging from 1 to 2, and it was not possible to conclude that IFX, Tac, and cyclosporine have comparable efficacy; second, the results from Komaki's study was conducted from indirect comparisons. There are 2 studies related to Tac comparing with placebo, 2 studies related to IFX comparing with placebo, but no studies related Tac comparing with IFX. Our meta-analysis, however, included 6 studies directly comparing Tac and IFX; therefore, the results from our study are more robust.

There are some limitations to the current meta-analysis. First, the number of included studies was relatively small and comprised only approximately 438 cases. Among the 6 included studies, only 1 study was an RCT, and the other 5 were retrospective studies. Therefore, the results from our meta-analysis need to be confirmed by large RCTs. Second, all of the included studies were conducted in Japan and the data from other countries and races are lacking; therefore, the interpretation of the results of the current meta-analysis should be taken with caution especially applying to western population. Third, the present meta-analysis did not compare the long-term efficacy and safety of Tac and IFX because of the limited data in this respect. Fourth, the results of this meta-analysis should be interpreted with caution given heterogeneity of patient population (steroid refractory UC, severe UC, and moderate to severe UC) and varying number of studies reporting various end points and the different criteria to define clinical response and remission.

In conclusion, our meta-analysis suggested that both Tac and IFX appeared to be effective and safe as rescue therapies for moderate-to-severe active UC and steroid-refractory UC; however, no definitive difference between Tac and IFX was demonstrated. Further studies comparing Tac and IFX are needed.

## Acknowledgments

The authors are particularly grateful to Dr Wang for his kind help in the final editing of this manuscript. The authors also thank ELIXIGEN for their English service.

## Author contributions

**Conceptualization:** Yi-Juan Liu, Cheng-Dang Wang.

**Data curation:** Yi-Juan Liu, Hua Fan, Wei-Wei Zhen.

**Formal analysis:** Yi-Juan Liu, Hua Fan, Wei-Wei Zhen, Xing Yu.

**Funding acquisition:** Cheng-Dang Wang.

**Investigation:** Jin-Tong Chen.

**Methodology:** Wei-Wei Zhen, Xing Yu.

**Project administration:** Jin-Tong Chen.

**Resources:** Jin-Tong Chen.

**Software:** Wei-Wei Zhen, Xing Yu.

**Supervision:** Cheng-Dang Wang.

**Validation:** Yi-Juan Liu, Hua Fan, Xing Yu, Jin-Tong Chen, Cheng-Dang Wang.

**Writing – original draft:** Yi-Juan Liu.

**Writing – review and editing:** Yi-Juan Liu, Cheng-Dang Wang.

## Supplementary Material

Supplemental Digital Content
